# Do Exogenous DNA Double-Strand Breaks Change Incomplete Synapsis and Chiasma Localization in the Grasshopper *Stethophyma grossum*?

**DOI:** 10.1371/journal.pone.0168499

**Published:** 2016-12-22

**Authors:** Adela Calvente, Juan Luis Santos, Julio S. Rufas

**Affiliations:** 1 Departamento de Biología, Edificio de Biológicas, Universidad Autónoma de Madrid, Madrid, Spain; 2 Departamento de Genética, Facultad de Biología, Universidad Complutense de Madrid, Madrid, Spain; University of California San Francisco, UNITED STATES

## Abstract

Meiotic recombination occurs as a programmed event that initiates by the formation of DNA double-strand breaks (DSBs) that give rise to the formation of crossovers that are observed as chiasmata. Chiasmata are essential for the accurate chromosome segregation and the generation of new combinations of parental alleles. Some treatments that provoke exogenous DSBs also lead to alterations in the recombination pattern of some species in which full homologous synapsis is achieved at pachytene. We have carried out a similar approach in males of the grasshopper *Stethophyma grossum*, whose homologues show incomplete synapsis and proximal chiasma localization. After irradiating males with γ rays we have studied the distribution of both the histone variant γ-H2AX and the recombinase RAD51. These proteins are cytological markers of DSBs at early prophase I. We have inferred synaptonemal complex (SC) formation via identification of SMC3 and RAD 21 cohesin subunits. Whereas thick and thin SMC3 filaments would correspond to synapsed and unsynapsed regions, the presence of RAD21 is only restricted to synapsed regions. Results show that irradiated spermatocytes maintain restricted synapsis between homologues. However, the frequency and distribution of chiasmata in metaphase I bivalents is slightly changed and quadrivalents were also observed. These results could be related to the singular nuclear polarization displayed by the spermatocytes of this species.

## Introduction

Meiosis determines the fate of chromosomes during the sexual life cycle by maintaining the chromosome number across generations in eukaryotes. It consists of two successive nuclear divisions preceded by one round of DNA replication and the formation of four haploid daughter cells. In most organisms, during the prophase of the first division, and after homologous chromosome recognition (pairing), homologous chromosomes suffer reciprocal recombination events (crossovers, COs), cytologically manifested as chiasmata. COs and the independent chromosome segregation generate novel combinations of parental alleles and boost the genetic diversity of meiotic products. Moreover, COs and sister chromatid cohesion are responsible for the correct bi-orientation of bivalents at metaphase I that leads to the segregation of a complete set of chromosomes at anaphase I. Thus, the chromosome complement is halved at the end of the first division. Sister chromatids separate at the second division generating four haploid meiotic products that originate the gametes. The fusion of gametes at fertilization restores the diploid chromosome number of the species [1].

Meiotic homologous recombination occurs through repair of programmed DNA double-strand breaks (DSBs) generated by SPO11, a topoisomerase II-like protein, and its accessory factors [2]. Then, SPO11 is endonucleotically excised from break ends so that one strand is resected from 5’ to 3’. Exposed single-stranded DNA regions search for and invade homologous DNA duplex with the help of recombinases RAD51 and DMC1. Then recombination intermediates will be resolved in different recombination products: COs that are characterized by the reciprocal interchange of flanking markers, and noncrossovers (NCOs) in which flanking DNA remains unchanged. In the majority of organisms the number of COs per bivalent is low when compared with the number of DSBs initially formed [3–5]. Thus, the majority of DSBs become NCO products. CO formation is tightly regulated and ensures that each bivalent has at least one CO, the obligate crossover, to promote the correct segregation of homologous chromosomes. In most organisms, COs preferentially occur at discrete sites called hotspots [6–10]. An extreme cytological manifestation of this situation is chiasma localization in which COs occur preferentially or exclusively in certain chromosome regions [11–15]. In order to understand the relationship between recombination and synapsis, exogenous DSBs have been induced by different methods such as ionizing radiation and chemotherapeutic agents, e.g., cis-platinium (II) diammine dichloride (cisplatin). For instance, Hanneman et al. [16] treated mice males with cisplatin to increase recombination. Males were then mated to monitor the number of COs inherited by the offspring. The CO frequency in the chromosomes analyzed was almost two times higher than that in untreated males. On the other hand, and for similar purposes, Storlazzi et al. [17] used γ rays in *Sordaria macrospora* and Sánchez-Morán et al. [18] employed cisplatin in *Arabidopsis thaliana*. In both cases, recombination, synapsis, and fertility were partially restored in *spo11* mutants. Analogous experiments in different species have revealed similar results [19–23].

**Fig 1:**
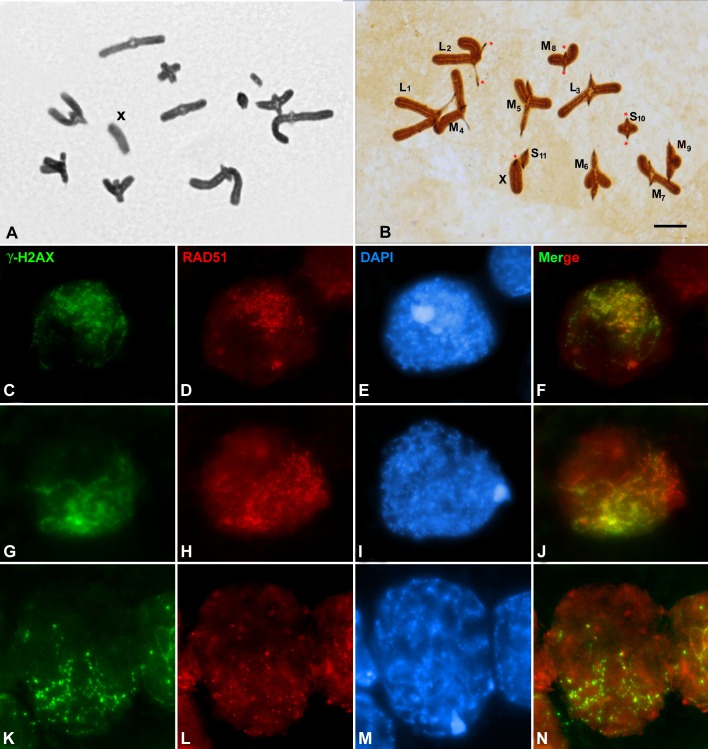
**Acetic orcein and silver staining of metaphase I spermatocytes (A, B). (C-N)** γ**-H2AX and RAD51 double immunolocalization in non-irradiated squashed spermatocytesof *S*.*grossum*.** (A) The X chromosome is marked. Most bivalents show a single chiasma located near the pericentromeric regions. (B) Kinetochores of chromosomes are marked by asterisks. See text for the nomenclature applied to different bivalents. (C,G,H) γ-H2AX (green); (D,H,L) RAD51 (red); (E,I,M) DAPI; (F,J,N) merging of γ-H2AX and RAD51. (C-F) Leptotene. (G-J) Zygotene. (K-N) Pachytene. γ-H2AX and RAD51 signals are polarized in the same nuclear region. (C-N) images correspond to the sum of different focal planes. Bars represent 10 μm.

Here we wondered whether exogenous DSBs could modify the frequency and distribution of COs in the grasshopper *Stethophyma grossum*. The karyotype of this species is composed of telocentric chromosomes (2n = 22+X in males, 22+XX in females). According to their size they have been divided into three groups: long chromosomes (L1-L3); medium chromosomes (M4 -M9) and short chromosomes (S10 and S11). Male meiosis is quite unusual: i) Bivalents are mostly monochiasmatic, although two chiasmata have occasionally been observed in the M9 bivalent. The single chiasma is not restricted to any particular region in the shortest bivalents (M9-S11), but it is exclusively located near the pericentromeric region in the rest of the bivalents (L1-M8) (Fig 1A and 1B; [24–26]. ii) Full synapsis is only achieved by the three shortest bivalents, whereas the remaining bivalents show incomplete synapsis restricted to pericentromeric regions [27–29]. In this species, as in other grasshoppers [30], the initiation of recombination, detected by γ-H2AX labelling, precedes synapsis and recombinase RAD51 foci appear after DSB formation [31].

## Materials and Methods

A total of 77 adult males of *Stethophyma grossum* (Orthoptera; Acrididae) were used in this study. They were captured in a natural population from Orea (Guadalajara, Spain).*Stethophyma grossum* is not an endangered species and captures were done under permission of the "Organismo Autónomo Espacios Naturales de Castilla-La Mancha" that is the responsible authority on Conservation under Spanish legislation.

### Irradiation with γ rays

We applied different doses of gamma irradiation (1Gy, 2.5Gy, 5Gy and 10Gy) to adult males to produce extensive exogenous non-programmed DSBs. We analyzed the meiotic chromosome behavior at different times after each irradiation experiment, namely: one hour, and daily from the first until the twelfth day in 52 samples. Doses higher than 5Gy produced total mortality of individuals in 1 or 2 days after irradiation. 2.5Gy and 5Gy treatments provoked alterations in the chiasma pattern (bivalents with two chiasmata and the appearance of quadrivalents). 50 spermatocytes were analyzed in each experiment. The alterations appeared in 23% (2.5Gy treatment) and 77% (5Gy treatment) of diakinesis-metaphase I spermatocytes. Obviously, these treatments surely produce persistent and unrepaired DNA damages that are eliminated through multiple meiotic checkpoints. However, our interest was focused on the changes that could be produced in the synapsis and recombination patterns of the surviving meiocytes. Hence, we considered that 5Gy was the most adequate dose for our purposes. The results obtained in this scenario came from the study of meiosis in 25 males.

### Acetic orcein staining

Testes were fixed in 3:1 ethanol:acetic acid and stored at 4°C until required. Two or three tubules and a drop of acetic orcein were placed per slide. Once the excess of dye was removed, a drop of 50% acetic acid was added and the material squashed [32]. The material was frozen in liquid nitrogen, air-dried and mounted with Eukitt.

### Silver staining

Testes were fixed in 3:1 ethanol–glacial acetic acid and stored at −20°C until required. Silver staining was carried out in single follicles that were squashed in a drop of 50% acetic acid, coverslips were removed after freezing in liquid nitrogen and slides were then air-dried. Slides were incubated in 2 X Saline–Sodium Citrate (SSC; 300 mM NaCl, 30 mM Na3C6H5O7, pH 7.0) at 60° C for 15–25 min, rinsed thoroughly in tap water and air dried. A drop of AgNO3 solution (0.1 g of AgNO3 in 0.1 ml of distilled water adjusted to pH 3 with formic acid) was placed on each slide, covered with a coverslip and incubated in a moist chamber at 80°C. After 3 min, the degree of staining was monitored under a light microscope. Finally, slides were rinsed in tap water, air-dried and mounted in Eukitt.

### Spermatocyte preparation for protein immunodetection

Testes were extracted and the seminiferous tubules placed in PBS (137 mM NaCl, 2.7mM KCl, 10.1 mM Na2HPO4, 1.7mM KH2PO4, pH 7.4) until their processing by either squashing or spreading. For the squashing of spermatocytes we have followed the technique previously described [33]. Briefly, seminiferous tubules were fixed for 10 min in freshly prepared 2% formaldehyde in PBS containing 0.1% Triton X-100 (Sigma). After 5 min, several seminiferous tubules fragments were placed on a slide coated with 1 mg/ml poly-L-lysine (Sigma) with a small drop of fixative, and gently minced with tweezers. The tubules were then squashed and the coverslip removed after freezing in liquid nitrogen. Spread preparations were obtained following the drying-down technique previously described [34]. Briefly, the apical region of the tubules was disaggregated in 200μl of 100 mM sucrose in distilled water and macerated at room temperature for 15min.Spermatocytes were simultaneously fixed and spread onto a slide with 1% paraformaldehyde in distilled water containing 0.15% Triton X-100 (Sigma, St. Louis, Missouri, United States). Preparations were air-dried during 2 h in a moist chamber and washed twice with 0.08% Photo-Flo (Kodak) in distilled water.

### Antibodies

A monoclonal mouse antibody (#05–636; Upstate) raised against amino acids 134–142 of human histone γ-H2AX was used to detect the histone variant γ-H2AX [35] at 1:500 dilution in PBS. This peptide sequence is identical in yeast and mouse (Redon et al., 2002). A polyclonal rabbit anti-RAD51 antibody (Ab-1; PC130; Calbiochem), generated against recombinant HsRAD51 protein, was used to detect the recombinase RAD51 at 1:30 dilution in PBS. The cohesin subunit SMC3 was detected by a polyclonal rabbit anti-SMC3 antibody (AB3914; Chemicon International) raised against a synthetic peptide from human SMC3 at 1:30 dilution in PBS. The cohesin subunit RAD21 was detected with a rabbit anti-RAD21 antibody raised against a bacterially expressed carboxyl-terminal fragment of *Drosophila melanogaster* RAD21 at 1:30 dilution in PBS (kindly provided by Dr. Margarete M.S. Heck from Centre for Cardiovascular Science, University of Edinburgh).

### Protein immunodetection

The slides of both squashed and spread spermatocytes were rinsed three times (each time for 5 min) in PBS. Spermatocytes were incubated overnight at 4°C with primary antibodies. After three washes of 5 min in PBS, primary antibodies were revealed with the appropriate secondary antibodies conjugated with either FITC or Texas Red (Jackson) at 1:150 dilution in PBS. In double immunolabelling of antibodies raised in different species, both primary antibodies were incubated overnight at 4°C simultaneously. Then, the samples were washed three times (each time for 5 min) in PBS and revealed with secondary antibodies. In double immunolabelling of antibodies raised in the same species, a primary antibody was added and incubated overnight at 4°C. Then, the sample was washed three times (each time for 5 min) in PBS and revealed with the appropriated secondary antibody FITC-conjugated IgG, F(ab’)2 (Jackson laboratories). After overnight incubation at 4°C the sample was washed three times in PBS, incubated with the second primary antibody overnight at 4°C, washed in PBS and revealed with the appropriated secondary antibody Texas Red-conjugated. Chromatin was counterstained with DAPI 10 μg/ml. The slides were thoroughly washed in PBS and finally mounted with Vectashield (Vector Laboratories). For the double-immunolabeling of SMC3 and RAD51 in which the primary antibodies were generated in the same host species, we proceed as previously described [36, 37]. The slides were initially incubated with the anti-SMC3 antibody for 1 h at room temperature, rinsed three times (each time for 5 min) in PBS and incubated overnight at 4°C with an FITC-conjugated goat Fab' fragment anti-rabbit IgG (Jackson) at 1:100 dilution in PBS. Subsequently, slides were rinsed at least six times (each time for 5 min) in PBS, incubated with anti-RAD51 for 1 h, rinsed three times (each time for 5 min) in PBS, and then incubated with a Texas Red-conjugated goat anti-rabbit IgG (Jackson) at a 1:150 dilution. Finally, all slides were rinsed three times (each time for 5 min) in PBS and counterstained for 3 minutes with 5μg/ml DAPI. After a final rinse in PBS, the slides were mounted in Vectashield (Vector Laboratories). Observations were performed using an Olympus BX61 microscope equipped with a motorized Z axis and epifluorescence optics. Stacks of images were captured with a DP70 Olympus digital camera using the AnalySIS software from Olympus and finally analyzed and processed using the public domain ImageJ software (National Institutes of Health, USA; http://rsb.info.nih.gov/ij), VirtualDub (VirtualDub.org; http://www.virtualdub.com) and Adobe Photoshop 7.0 software.

### Distance measurement between homologous telomeres

This analysis was performed in selected chromosomes of late zygotene nuclei stained with antibodies against the SMC3 subunit that are presumably related to the development of the axial element (AE) of the synaptonemal complex (SC). Photographs at different planes separated 0.2μm each one, were taken to estimate the distance between unsynapsed homologous telomeres. The number of planes between the focused telomeres multiplied by the inter-plane distance provides the vertical distance between the unsynapsed telomeres. 2D projections of all planes corresponding to the same nucleus allow the estimation of the horizontal inter-telomere distance.

## Results

### Background

During prophase I, spermatocytes of *S*. *grossum* show a remarkable nuclear polarization of the axis maturation of cohesin subunits, and the location of γ-H2AX, which marks the sites of double-strand breaks, and RAD51, which is involved in homology search. This polarization was observed in both squashed and spread spermatocytes [31, 38, 39], and is maintained throughout prophase I along the chromosome regions that either are undergoing or are about to undergo synapsis (Fig 1C–1N). The identification of different early prophase I stages was carried out according to the degree of synapsis displayed by the shortest chromosomes and the chromatin morphology. According to these observations, it was proposed that the restricted distribution of recombination events along the chromosomal axes is responsible for the incomplete presynaptic homologous alignment and, hence, for the partial synaptonemal complex formation displayed by most bivalents[31, 38]. This proposal did not exclude the possibility that these processes may also be influenced and modulated by changes in chromatin organization.

### Distribution of γ-H2AX and RAD51 at prophase I of irradiated males

**Fig 2:**
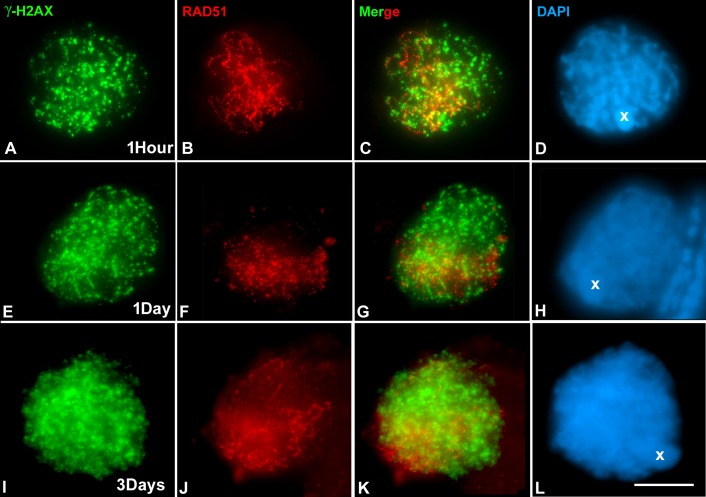
Double immunolocalization of γ-H2AX (green) and RAD51 (red) in squashed spermatocytes of *S*. *grossum* irradiated with 5Gy dose of gamma rays. The chromatin is counterstained with DAPI (blue). (A-D) Late leptotene. Immunolocalization of γ-H2AX and RAD51 one hour after irradiation. Whereas γ-H2AX labelling is distributed in the whole chromatin, RAD51 foci are concentrated in a nuclear region. (E-H) Zygotene. One day after treatment. The labelling of γ-H2AX covers the whole nucleus but that of RAD51 is polarized in a nuclear area. (I-L) Late zygotene. Three days after treatment. The distribution of γ-H2AX and RAD51 is maintained. All images are the sum of all focal planes of a nucleus. Bar represents 10 μm.

Results obtained in experiments with different doses of γ rays (see Materials and Methods section) indicated that 5Gy was the most appropriate dose to induce a high level of exogenous DBSs that was compatible with the survival of irradiated males. One hour after 5Gy treatment, γ-H2AX foci were uniformly distributed at late zygotene nuclei (n = 50) although the polarization of RAD51 signals was evident (Fig 2A–2D; S1 Video). This situation was maintained in samples taken at 5h (data not shown) and one day after treatment (Fig 2E–2H). In cells observed after three days, marks of DNA damage were maintained in the whole nucleus but RAD51 foci were mostly restricted to a nuclear region corresponding to the paired cohesin axes (n = 70; Fig 2I–2L). However, about 15% of RAD51 signals were dispersed over the nucleus. These may be related to episodes of recombination between sister chromatids and, to a lesser extent, CO events.

### Full synapsis is not achieved after irradiation

**Fig 3:**
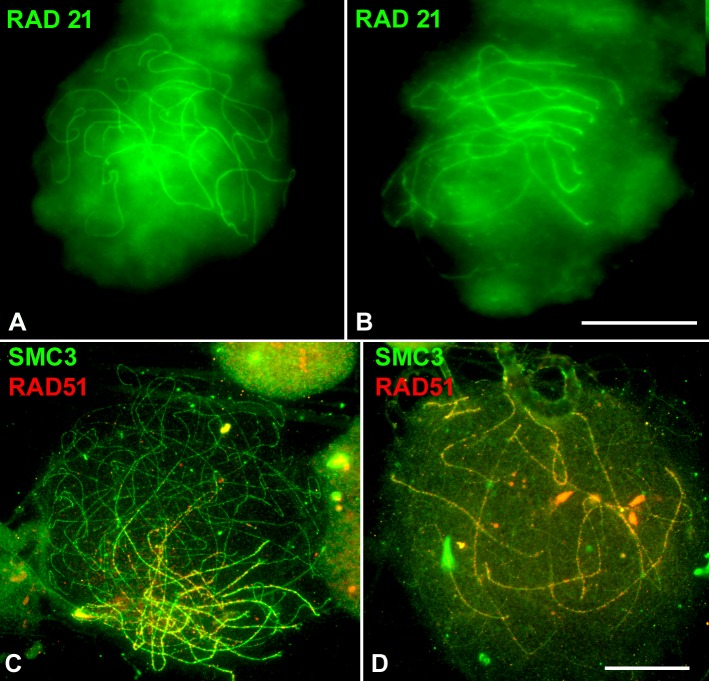
**Pairing and recombination in non-irradiated (A, C) and irradiated (B, D) *S*. *grossum* spermatocytes.** (A, B) Polarized RAD21 axis labelling (green) in squashed late zygotene nuclei. (C-D) Double immunolocation of SMC3 (green) and RAD51 (red) in spread late zygotene nuclei. (A, B). (C, D) SMC3 paired cohesin axes are polarized despite the possible disturbances produced by the spreading procedure. RAD51 signals are almost absent in the unsynapsed chromosome regions of both non-irradiated (C) and irradiated (D) spermatocytes. A and B images are the sum of all the photographs taken at different focal planes and are at the same magnification. C and D are at the same magnification. Bars represent 10 μm

Since in this species it is not possible to assay directly the synaptonemal complex (SC) formation, we have inferred it from the identification of cohesin subunits (SMC3 and RAD21). These proteins are located at the base of chromatin loops and may be involved in the assembly of the axial/lateral elements of SC. Thus, SMC3 paired cohesin axes that form thick filaments would indicate synapsed regions while thin axes would represent unsynapsed regions [40]. However, RAD21 would only be located in synapsed regions. Two and three days after irradiation, no alteration was observed in the RAD21 labelling pattern with respect to that of non-irradiated individuals (Fig 3A and 3B). Furthermore, the co-immunolocalization of RAD51 and SMC3 was also maintained in both situations (n = 20; Fig 3C and 3D).

### Metaphase I observations

**Fig 4:**
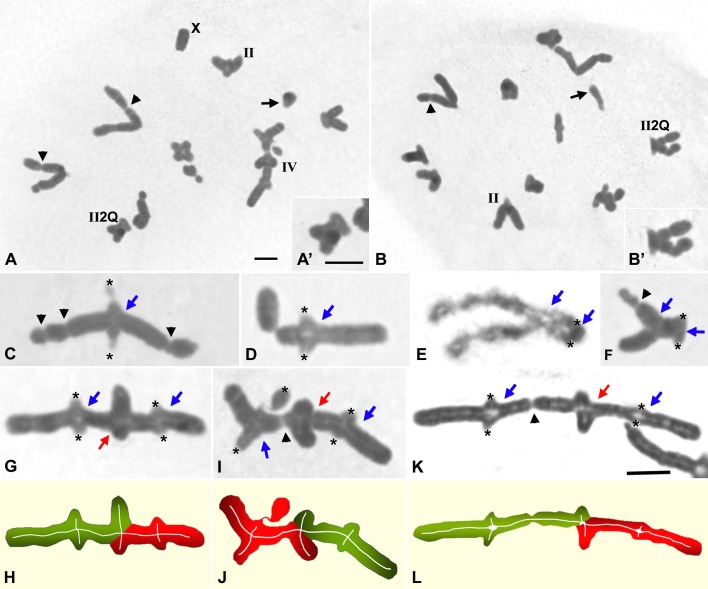
Acetic orcein staining of squashed metaphase I chromosomes of *S*. *grossum* males 5–6 days post-irradiation. Black arrowheads indicate chromosome fragments and secondary constrictions. (C-K) Centromere positions are indicated by black asterisks. Blue arrows mark the position of a chiasma between homologous chromosomes. (G-K) Red arrows indicate the chiasma joining non-homologous chromosomes. (A) Metaphase I. The single X chromosome, a bivalent with a single chiasma (II), a quadrivalent (IV) and a bichiasmatic long bivalent (II2Q) are indicated. (B) Metaphase I. A monochiasmatic bivalent (II) and a bichiasmatic bivalent (II2Q) are indicated. (A’, B’) Enlargements of the bichiasmatic bivalents of A and B respectively. (C, D) Bivalents with a single proximal chiasma. (E, F) Bichiasmatic bivalents at diakinesis (E) and metaphase I (F). Positions of chiasmata are indicated by blue arrows. (G, I, K) Quadrivalents. (H, J, L) Diagrams of quadrivalents shown in G, I and K. Homologous chromosomes are shown in red and green, respectively. A and B, A’ and B’ and C-K have the same magnification. Bars represent 10 μm

To study the effect of 5Gy dose on the frequency and distribution of chiasmata, we analyzed male meiosis throughout the period corresponding to 1–12 days after irradiation. Most diakinesis-metaphase I chromosomes presented chromatin constrictions and breakages as a consequence of the DNA damage produced by irradiation (black arrowheads in Fig 4). In diakinesis-metaphase I cells (n = 75) of males sacrificed 5–6 days after irradiation,we only analyzed morphologically undamaged bivalents, i.e., those without any sign of fragmentation. Thus, we identified 202 L1-M8 bivalents with a single chiasma located near the pericentromeric region, and 30 bichiasmatic bivalents: 23 corresponding to L1-M8 chromosomes and 7 to M9 chromosomes (Fig 4A, 4A’, 4B, 4B’, 4E and 4F). In bichiasmatic L1-M8 bivalents, one chiasma was always located near the pericentromeric region while the other one was invariably located in an interstitial position (white arrows in Fig 4E and 4F). Moreover, we scored up to 19 quadrivalents in which two long bivalents were invariably joined by three chiasmata (Fig 4A and 4G–4L).

### Anaphase I and second division observations

**Fig 5:**
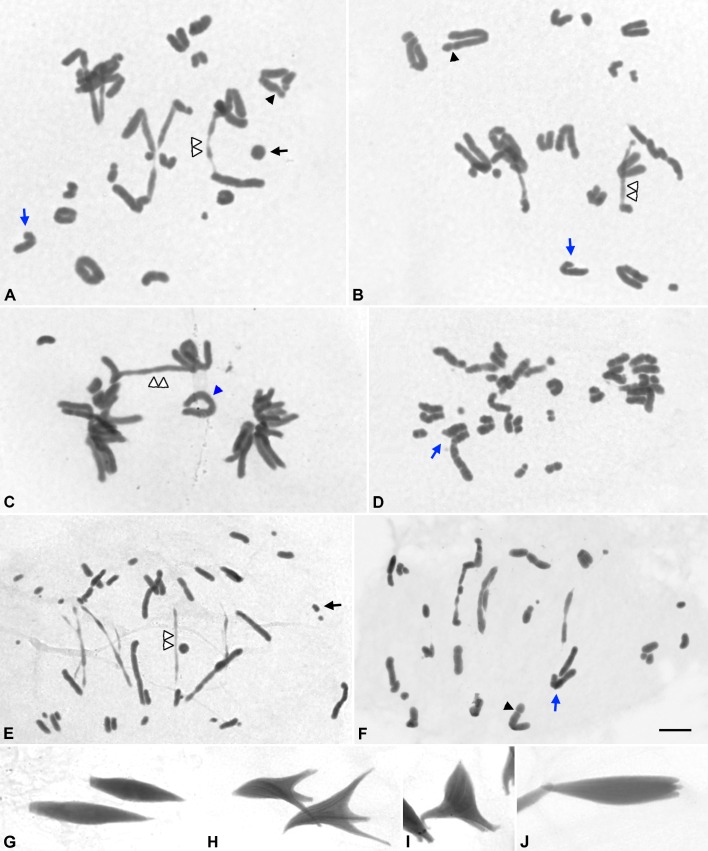
Acetic orcein staining of squashed spermatocytes of *S*. *grossum* at anaphase I and second meiotic division, 8–12 days after irradiation. (A, B) Anaphase I. (C) Telophase I. (D) Metaphase II. (E,F) Anaphase II. (G) Haploid spermatids. (H-J) Aberrant spermatids. Chromatin bridges (double white arrowheads), chromosomal fragments (black arrows), chromatin constrictions (black arrowheads), heteromorphic chromosomes (blue arrows) and lagging chromosomes (blue arrowhead) are indicated. Bars represent 10 μm.

Seven days after irradiation, several unusual chromatin configurations were commonly observed: metaphase II-like configurations with aberrant number of chromosomes, anaphase I and anaphase II cells containing chromosome laggards and chromatid bridges (Fig 5A–5F).

Consequently, there was a significant presence of abnormal spermatids with different ploidy levels (Fig 5G–5J). From the day eight up to the day twelve after irradiation, the number of chromosome/chromatid fragments, secondary chromatin constrictions and chromosome fusions increased considerably. Chromosome entanglements were predominant in most of the cells, preventing cytological analysis (S1 Fig).

### Nuclear organization of *Stethophyma grossum* spermatocytes

**Fig 6:**
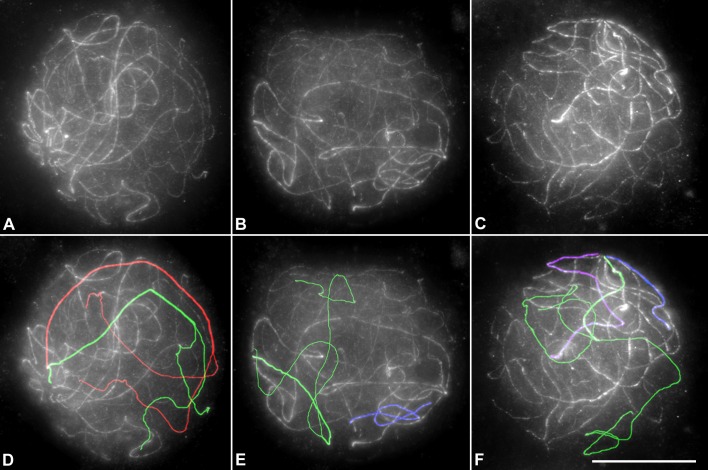
Nuclear organization. Projection of several focal planes of spermatocytes of *S*. *grossum* in which SMC3 has been located. Drawings of some bivalents have been superimposed in D, E and F. Notice that the homologous telomeric ends of long bivalents remain at the nuclear periphery. The separation between the unsynapsed ends is evident even in a Z projection (see red and green bivalents in D, E and F, and also S2 and S3 Videos). In E and F, short full synapsed bivalents are drawn in blue and purple. Bar represents 10 μm.

At this point, we wondered whether the results described above could be related to possible differences in the nuclear organization of irradiated and non-irradiated spermatocytes. To do that, chromosomes were stained with an antibody against the SMC3 cohesin subunit and their nuclear distribution analyzed at late zygotene. In non-irradiated spermatocytes we identified two different chromosome regions in L1-M8 bivalents at zygotene, namely: those with partial homologous synapsis near the pericentromeric regions, and those that are unsynapsed and do not exhibit any type of alignment. These regions show the axes separated (mean distance of 8.7 μm in n = 20) (Fig 6; S2 and S3 Videos). This spatial chromosome organization was also maintained in irradiated spermatocytes three days after treatment.

## Discussion

### Distribution of γ-H2AX and RAD51 in prophase I spermatocytes of irradiated males

It has been reported that irradiation with gamma rays produces exogenous DBSs that lead to changes in chiasma frequency [17]. On the other hand, studies on RAD51 kinetics have revealed that 80% of DSBs are repaired 30h after irradiation in mice meiosis [19], and that 60% of human fetal glial cells present RAD51 foci six hours after irradiation [35]. On these grounds, and to be confident in the dynamics of DNA repair process in our experiment, we decided to analyze the distribution of SMC3, RAD21, γ-H2AX and RAD51 in males sacrificed during the first three days after 5Gy irradiation. Whereas γ-H2AX labelling, indicative of the DNA damage produced, occupied the whole prophase I nuclei, RAD21 mature filaments and RAD51 foci showed a broad polarized distribution. Therefore, in contrast to what is observed in non-irradiated spermatocytes, RAD51 is not recruited to all the regions where exogenous DBSs have been produced (Figs 2 and 3, S1 Video). This scenario contrasts with that of non-irradiated spermatocytes where these proteins show nuclear polarization (Fig 1C–1N). RAD51 is only recruited in those regions where programmed DSBs have been produced. Chromatin configuration and correct formation of both cohesin axes and axial elements (AEs) of chromosomes could be involved in the behavior of RAD51[17, 41–43]. Indeed, it has been proposed that the cohesin axis is either an integral part of the AE or the base upon the AE is built [41, 44, 45]. It is also known that meiotic repair proteins are associated with the axial/lateral elements of SC [46–48]. On these grounds, chromatin remodeling complexes and histone modifications may also play a role in the polarized distribution of RAD51 because the former are indispensable factors for DNA repair, and may unravel the chromatin packaging needed to facilitate the access of repair enzymes to regions of DNA damage [49, 50]. Moreover, in other orthopteran species in which homologous chromosomes show incomplete synapsis and chiasma localization, there is also a conspicuous polarization of both the maturation of cohesin axes and the initiation of meiotic recombination events [12, 34]

### Minor changes in chiasma localization despite massive formation of DSBs

**Fig 7:**
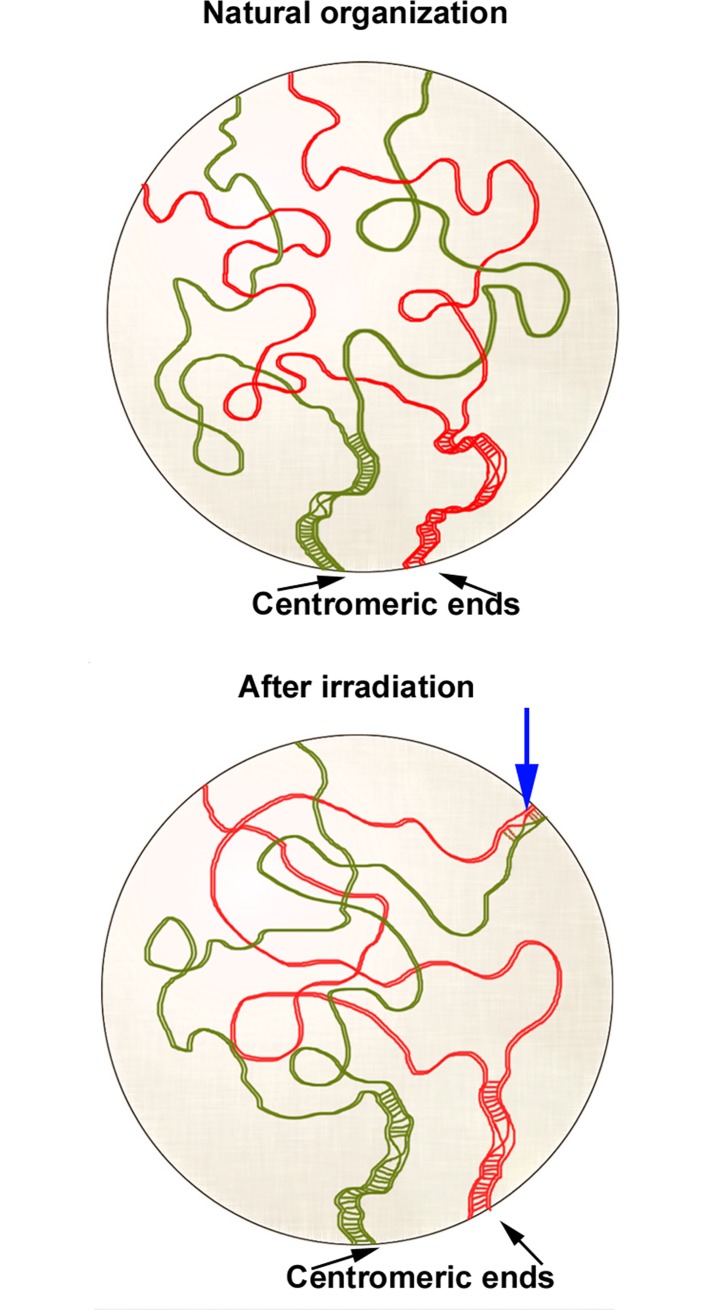
Possible origin of quadrivalents. Diagramatic representation of two long bivalents, partially synapsed, of *S*.*grossum*, depicted in green and red, respectively. Whereas chiasmata normally occur in the pericentromeric regions of homologous chromosomes, they could also take place between homologous subdistal regions of non-homologous chromosomes as a consequence of reciprocal translocations produced by exogenous DSBs and /or COs between homologous DNA sequences of non-homologous chromosomes (non-allelic homologous recombination) (blue arrow).

Whereas paired SMC3 and RAD21 stretches near the pericentromeric regions are observed at late zygotene spermatocytes of both irradiated and non-irradited individuals homologous unpaired cohesin axes are spatially far apart (Fig 6; S2 and S3 Videos). This peculiar nuclear chromosome disposition and, likely, the chromatin organization in unsynapsed chromosome regions are factors that could be involved in the strict localization of the single chiasma at the pericentromeric regions of L1-M8 bivalents [38, 39]. Despite the high increase of exogenous DNA DSBs originated by irradiation, cytologically manifested by the γ-H2AX distribution in the whole nuclei, only 13% of the metaphase I bivalents analyzed (30/232) showed two chiasmata (Fig 4A, 4A’, 4B, 4B’, 4E and 4F). The additional chiasma was always located in the middle of the chromosome (Fig 4G and 4L). Therefore, one could confidently propose that these extra COs could be originated by the exogenous DBSs produced in those chromosome regions in which a polarization of both cohesin axes and recombination machinery still remain. On the other hand, exogenous DSBs could originate quadrivalents by either reciprocal translocations or COs between homologous DNA sequences of non-homologous chromosomes (non-allelic homologous recombination) (Figs 4H–4L and 7).

In summary, we have tried through the induction of exogenous DSBs to alter synapsis and recombination patterns in a species in which most of the chromosomes display incomplete synapsis and proximal chiasma localization. However, we have not found a significant increase of COs. perhaps because that the particular model of recombination of this species is under a very strict control, probably based on the peculiar nuclear chromosome spatial organization and the local differences in chromatin configuration.

## Supporting Information

S1 Fig Acetic orcein staining of *S*. *grossum* spermatocytes 6–12 days after 5Gy irradiation. (A, E, G) Diplotene. (G) Diakinesis (B, F, H-L) Metaphase I. (C) Anaphase I with chromatid bridges (white arrowheads). (D) Telophase I. The time of fixation after irradiation (days) is shown in all pictures. Bar represents 10 μm.
(TIF)

S1 Video Double immunolocation of γ-H2AX (red) and RAD51 (green) in *S*. *grossum* late zygotene spermatocyte. This video corresponds to the 3-D reconstruction from 70 focal planes of a late-zygotene spermatocyte fixed one day after 5Gy irradiation. Chromatin is DAPI stained. Notice that whereas γ-H2AX foci are distributed throughout the whole nucleus, RAD51 signals are restricted to a discrete nuclear region which corresponds to the synapsed regions of autosomal bivalents. See text for details. For the correct visualization of the video, please click the loop/bucle option in your video player before running the video.
(AVI)

S2 Video The pattern of synapsis in a *S*. *grossum* spermatocyte. 3D reconstruction of 50 focal planes of a late zygotene nucleus in which SMC3 (green) has been located. Synapsed and unsynapsed chromosomal regions correspond to thin and thick SMC3 filaments, respectively. Trajectories of the filaments of two partially synapsed long bivalent homologous chromosomes are drawn in red and green. Notice the peripheral position of their distal, unsynapsed telomeric regions and the actual distance of these ends in a 3D situation. For the correct visualization of the video, please click the loop/bucle option in your video player before running the video.
(AVI)

S3 Video The pattern of synapsis in *S*. *grossum* spermatocytes. 3D reconstruction of 50 focal planes of a late zygotene nucleus in which SMC3 (grey) has been located. Synapsed and unsynapsed chromosomal regions correspond to thin and thick SMC3 filaments, respectively. Trajectories of the filaments of two partially synapsed long bivalents are drawn in red and green. Notice the peripheral position of their distal, unsynapsed telomeric regions and the actual distance of these ends in a 3D situation. For the correct visualization of the video, please click the loop/bucle option in your video player before running the video.
(AVI)
