# Supplementary figures and images for: Do Exogenous DNA Double-Strand Breaks Change Incomplete Synapsis and Chiasma Localization in the Grasshopper Stethophyma grossum?
Source: PLoS One. 2016 Dec 22;11(12):e0168499. doi: 10.1371/journal.pone.0168499 (PMC5179137; doi:10.1371/journal.pone.0168499)

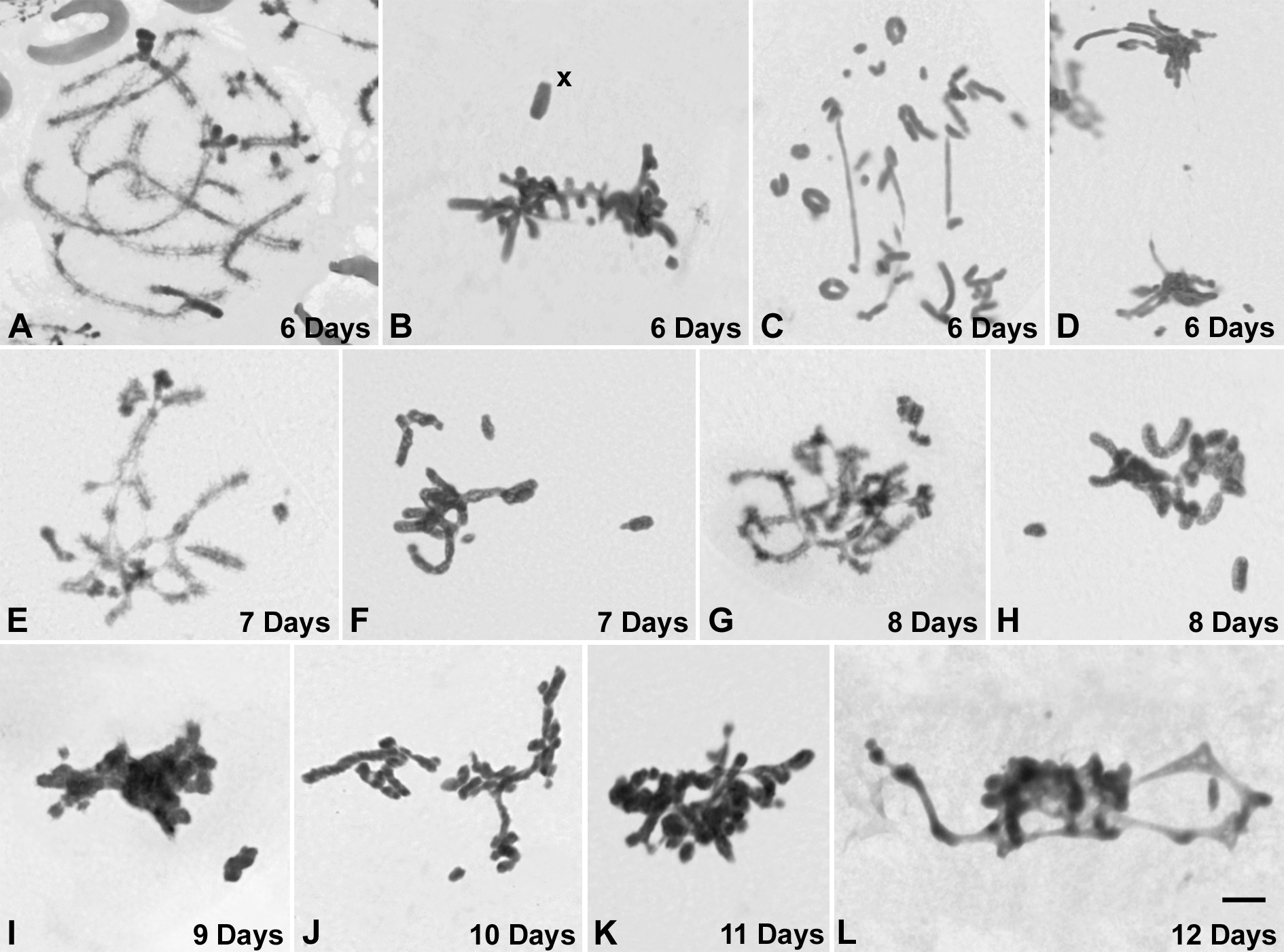

Supplement: S1 Fig — (A, E, G) Diplotene. (G) Diakinesis (B, F, H-L) Metaphase I. (C) Anaphase I with chromatid bridges (white arrowheads). (D) Telophase I. The time of fixation after irradiation (days) is shown in all pictures. Bar represents 10 μm. (TIF) [file pone.0168499.s001.tif]
